# MONDEP: A unified SpatioTemporal MONitoring Framework for National DEPression Forecasting

**DOI:** 10.1016/j.heliyon.2024.e36877

**Published:** 2024-08-28

**Authors:** Tipajin Thaipisutikul, Pasinpat Vitoochuleechoti, Papan Thaipisutikul, Suppawong Tuarob

**Affiliations:** a Faculty of Information and Communication Technology, Mahidol University, Nakhon Pathom, Thailand; b Department of Psychiatry, Faculty of Medicine Ramathibodi Hospital, Mahidol University, Bangkok, Thailand

**Keywords:** Depression forecasting, Machine learning, Deep learning, Time series forecasting

## Abstract

Depression has become a prevalent mental disorder that significantly affects a person's emotions, behaviors, physical health, ability to perform daily tasks, and ability to maintain healthy relationships. Untreated depression can escalate the risk of suicide, making the situation even worse. Despite an abundance of models previously proposed for forecasting depression, the issue of foretelling the overall number of patients at each administrative level remains under-investigated. Therefore, in this paper, we propose a simple but effective SpatioTemporal Monitoring Framework for National Depression Forecasting (MONDEP). In particular, we analyze national depression statistics data in Thailand as a case study and create prediction models for a real-time depression forecasting system using machine learning and deep learning approaches. In order to forecast the prevalence of depression at various administrative levels, we use the hierarchical structure of depression aggregation. The proposed framework consists of three modules: Data Pre-processing to extract and pre-process the raw data, Exploratory Data Analysis (EDA) to visualize and analyze the data to get insight, and Model Training and Testing to predict future depression cases. The objective of our research is to construct a comprehensive MONDEP framework that utilizes machine learning and deep learning to predict depression profiles at the district and national levels using multivariate time series across various administrative levels. Our study illustrates the considerable association between a spatial-temporal component and demonstrates how depression profiles may be represented by employing lower administrative-level data to estimate the general level of mental health across the nation. Additionally, the best performance across all criteria is obtained when a deep learning model is used to exploit multivariate time series, showing a 13% improvement in MAE measure compared to the SARIMAX baseline. We believe the proposed framework could be used as a point of reference for decision-making regarding the management of depression and has the potential to be incredibly helpful for policymakers in successfully managing mental health services on time.

## 1 Introduction

These days, mental health issues are a major national and international concern, particularly in the years after an epidemic. Depression is one of the most prevalent mental problems affecting individuals today. It is a severe and widespread medical condition that detrimentally impacts a person's feelings, thoughts, and actions. Depression [1], [2] may substantially negatively impact a person's ability to carry out daily chores, interpersonal connections, and quality of life. It significantly affects global impairment and, if untreated, can increase the risk of suicide.

The effects of mental health issues extend beyond a single person or family to encompass whole societies [3], [4]. Mental health problems have a detrimental effect on the quality of life, healthcare expenditures, and productivity. Additionally, they could breed prejudice and social stigma, which makes it harder for people to ask for assistance and support. It has also been discovered that marginalized and at-risk groups, such as minorities, refugees, and low-income people, are disproportionately affected by mental health issues and may have more challenges getting access to mental health treatments and assistance.

While numerous nations have established health service units specifically designed to assist individuals with mental health issues, the number of such units may be insufficient [5], [6], [7]. Several potential factors, including inadequate funding, labor scarcity, and institutional barriers to treatment access, may contribute to this unpredictability. Governments and health systems worldwide should therefore prioritize efforts to improve the forecasting of future national depression cases so that mental health treatment availability and accessibility can be more effectively planned.

Currently, several studies [8], [9], [10] have explored various factors associated with depression, including age, gender, socioeconomic status, and environmental factors such as weather patterns and geographic location, to predict depression rates in populations. Notably, our study is the first to exploit national statistics data, analyzed through machine learning algorithms [11], [12] to develop predictive models for depression rates. This innovative approach aims to identify populations at high risk of depression, providing crucial insights for policymakers in developing targeted prevention and intervention programs. However, there are still issues with utilizing national statistics to estimate depression rates due to the completeness of the data and the techniques that are used to deal with complementary signals among multivariate time series from spatial-temporal data for the following reasons: **1) Different administrative levels have different requirements for depression forecasting.** A more detailed understanding of the depression trend, supported by county-level forecasts, is also essential for the district government to coordinate district-level hospitalization and medical resources accordingly. For instance, the government needs district-level forecasts to estimate the future depression profiles of each district and make resource allocations across districts. However, predicting depression profile is a challenging task since the depression data across different administrative levels reveal extremely distinct dimensions and patterns; **2) Because the number of depressions in a particular location is influenced by the neighboring areas, forecasting is made considerably more challenging.** For instance, the future trend in depression in this area is influenced by both the local prevalence of mental disease and the adjacent regions. Moreover, these spatial correlations seem to be non-stationary and may be masked by unimportant noise, necessitating the dynamic excavation of useful mutual influence signals. Unfortunately, the majority of existing spatial-temporal methods [13], [14], [15], [16], [17] cannot effectively handle these enormous and complicated historical patterns to provide consistent and reliable forecasts without taking into account all of these difficulties and characteristics. To the best of our knowledge, no existing work focuses on the problem of spatiotemporal national depression forecasting at the city, district, and national levels of hierarchical granularity.

**Figure 1:**
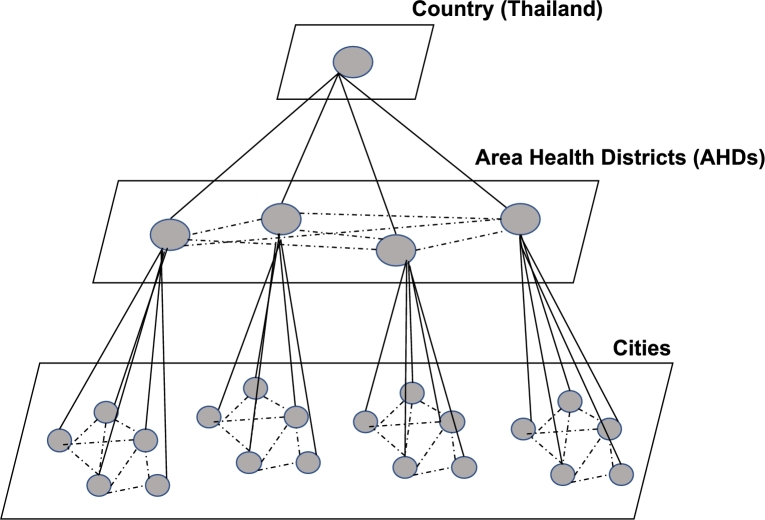
An illustration of the hierarchical perspective for depression profile modeling, where the dashed lines represent potential interactions between depression profiles within the same area and the solid lines represent the link between various administrative levels.

In this study, we propose a unified Spatiotemporal MONitoring Framework for National DEPression Forecasting, namely MONDEP, to facilitate joint learning of the number of national depressions across Area Health Districts (AHDs) of locations at different administrative levels and times. As depicted in Fig. 1, we utilize state-of-the-art machine learning and deep learning techniques to investigate the efficacy of national depression forecasting with a hierarchical spatial-temporal design. The depression aggregation follows a bottom-up hierarchical structure based on administrative topology, starting at the city level and then combined at the district and national levels. Fig. 1 provides an intuitive illustration of this hierarchical approach, where solid lines denote relationships between administrative entities, while dashed lines within each administrative level indicate potential depression-related interactions between intra areas.

The MONDEP is composed of three modules: 1) Data Pre-processing to standardize the raw data into the correct format; 2) Exploratory Data Analysis (EDA) to mine the relationship between depression profiles at lower levels and the national-level mental health status and find the hidden relations among the time series data; and 3) Model Training and Testing to determine the most effective model for predicting the future national depression cases.

Unlike previous studies that focus on identifying mental health symptoms at the individual level, our study emphasizes population-wide mental health monitoring at the district and national aggregate levels. We discovered that data in nearby regions are key indicators of accurate estimation of depression numbers in some areas. Additionally, the use of deep learning models in our research allows for more accurate predictions with less historical data. This shift to a broader, population-level perspective is crucial for advancing public health efforts and enhancing strategies for population-based depression prevention and treatment.

The main contributions of our study are summarized as follows.

• We offer a distinctive unified hierarchical approach for modeling national depressions, exploiting multi-variate time series for cooperative learning at different administrative levels.
• We propose an end-to-end machine-learning and deep-learning MONDEP framework to capture the dynamic and volatile spatial and temporal relationships related to depression profiles' forecasting at the district and national levels.
• We provide the MONDEP framework as a decision-support tool to foresee depression trends. The assessment's results demonstrate that deep learning models applied to exploit multi-variate time series data result in the most accurate projections.
• We also offer in-depth descriptive and statistical analysis of the relationship of the primitive factors regarding the dynamic surge of a mental-health illness.
• We make the source code and experimental results available for research purposes at.^1^

The rest of this work is structured as follows. Existing works relevant to our work are presented in Section 2, and the methodology is discussed in Section 3. Then, in Section 4 and 5, we provide experimental settings and results. Section 6, 7 and 8 provide the discussion on various aspects. Lastly, Section 9 summarizes the closing comments.

## 2 Related work

This section provides a comprehensive review of the current literature in two significant domains: forecasting of current trends in mental health illnesses, and developments in time series forecasting techniques.

### 2.1 Mental health illness forecasting

To address the research gap, we examined artificial intelligence in the context of mental illness by analyzing data types, modeling approaches, and current research trends. First, research on automated epidemiological surveillance from electronic health records (EHRs) is explored by [18], [19], [14]. The authors obtained 76% accuracy in the automatic extraction of autistic spectrum disorder (ASD) criteria from electronic health records (EHRs). The authors also utilized a traditional technique, such as topic modeling, for detecting suicide attempts in EHRs by recognizing certain signs, such as consistency in routine, emotional and social reciprocity, and nonverbal conduct. In addition, [20], [21] accurately identified post-traumatic stress disorder (PTSD) and schizophrenia by examining the writings submitted by patients. Also, the authors could classify the severity of schizophrenia patients' symptoms into four categories: absent, mild, moderate, and severe, using statistical analysis from textual information. Other studies [22], [23] found that traditional Machine Learning (ML) and Natural Language Processing (NLP) methods performed admirably but not always better than a professional's ability to predict clinical suicide risk in their patients. As a result, the authors recommended statistical NLP approaches to be used in conjunction with clinical practice. Furthermore, [24] proposed the comparison of regression and machine learning methods in depression forecasting. This study evaluated the effectiveness of regression versus machine learning methods in predicting depression among elderly Chinese. The findings suggested that machine learning methods may provide more accurate predictions by effectively capturing complex patterns in the data. [25] used machine learning-based prediction models for depression symptoms in Chinese healthcare workers during the early COVID-19 outbreak, highlighting that machine learning models can effectively predict depression symptoms. It underscored the importance of using these predictions to guide psychological interventions and support mental health management during public health emergencies. [26] reviewed current machine and deep learning techniques used for diagnosing various mental health conditions including depression. It provides a critical assessment of the methodologies used in recent studies and discusses the challenges and future directions for research in this area. [27] reviewed different machine learning algorithms used to diagnose depression, presenting a general model involving data extraction, prepossessing, and performance evaluation. It also outlines the future possibilities for research in the field of depression diagnosis using machine learning. [28] compared single classifier and ensemble machine learning approaches for predicting mental health issues, finding that ensemble methods may offer better performance due to their robustness against overfitting and ability to generalize across diverse data sets.

### 2.2 Time series forecasting

Another field with a long history is time-series forecasting, which has a wide range of classical methods, from traditional statistical methods like exponential smoothing and auto-regressive integrated moving average called ARIMA [29] to more recent hybrid methods [30], [31] that combine statistical methods with boosted trees [32], [33] or recurrent neural networks. The most recent pure deep learning solution is N-Beats [34]. On the other hand, pure time-series models solely consider changes in the temporal dimension while neglecting potential interactions between nodes in the geographical region.

The term spatial-temporal forecasting, which also refers to time-series forecasting with spatial interactions [35], [36], is used to describe a technique that may be used to anticipate traffic patterns and calculate travel times, among other things. With the aid of spatial analytic methods, researchers sought to identify the variables influencing the pandemic's spread. Spatial analysis has also been used in earlier research to examine and evaluate the spread of a number of illnesses, including dengue, cholera, diabetes, cancer, and flu viruses [37], [38], [39]. For instance, according to [39], local response tactics were exceedingly successful in stopping the spread of SARS, and that successful local management stopped the outbreak. These studies carried out at various scales and for various diseases, emphasize the consequences of spatial dependency across areas and help explain some of the regional variability of the transmission of diseases [38]. Several academics have looked at the existence of spatial effects for pandemic predictions today based on the outcomes of such studies. In this regard, comprehending the geographical distribution of Covid-19 is essential for both the creation of public health policies pertaining to the spread of early Covid-19 and for the epidemic prediction [39].

In addition, spatial-temporal analysis has been exploited in various domains such as location-based recommendation [40], and crime analytics in developing countries. For example, the authors [41], [42], [43], [44], [45] emphasized the detrimental impact of crime on economic growth and citizen well-being by proposing predictive analytics ARIMA model for time series prediction to address crime. The contribution lies in classifying crime patterns based on geographic density, identifying crime hotspots, and providing interactive visualization tools to aid law enforcement agencies in predicting and reducing crime occurrences.

Our research stands out as the first to conduct a spatial-temporal analysis of Thailand's depression patterns, focusing on a unique hierarchical data accumulation process. Moving beyond the individual-level focus of previous studies, we introduce a novel hierarchical approach for modeling depression profiles at district and national levels. This method offers a groundbreaking perspective that extends beyond individual analysis and marks a pioneering exploration into the nation's hierarchical data structure in mental health. Table 1 contains a summary of the existing models.

**Table 1:** Summary of key findings in related works.

Category	Key Findings	References
Mental Health Illness Forecasting	Automated epidemiological surveillance from EHRs; traditional techniques for detecting suicide attempts; identification of PTSD and schizophrenia from patient writings; classification of schizophrenia severity; ML and NLP methods for clinical suicide risk prediction.	[14], [18], [19], [20], [21], [22], [23], [24], [25], [26], [27], [28]

Time Series Forecasting	Classical methods like ARIMA; hybrid methods combining statistical methods with boosted trees or RNNs; N-Beats for pure deep learning solutions; spatial-temporal forecasting for pandemic spread analysis and other applications.	[29], [30], [31], [32], [33], [35], [36], [37], [38]

Spatial-Temporal Analysis	Used in traffic pattern prediction, crime analytics, and location-based recommendations; ARIMA model for crime prediction; identifying crime hotspots and providing visualization tools for law enforcement.	[40], [41], [42], [43], [44], [45]

## 3 The proposed framework: MONDEP

**Figure 2:**
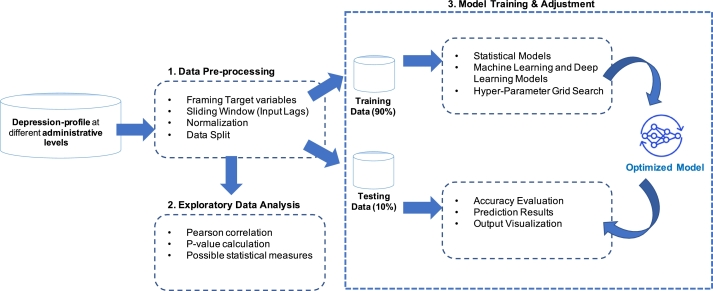
A High-level Diagram of the proposed MONDEP framework. MONDEP consists of three modules: 1) Data Pre-Processing Module; 2) Exploratory Data Analysis (EDA) Module; 3) Model Training and Testing Module.

In this section, we provide further information on MONDEP, our unified spatiotemporal monitoring system for predicting national depression in Thailand. Its main architecture is depicted in Fig. 2. We first discuss the preliminary steps of data pre-processing in Section 3.1. Exploratory Data Analysis (EDA) and the model training and testing module for MONDEP are then presented in Sections 3.2 and 3.3, respectively.

### 3.1 Data pre-processing

**Figure 3:**
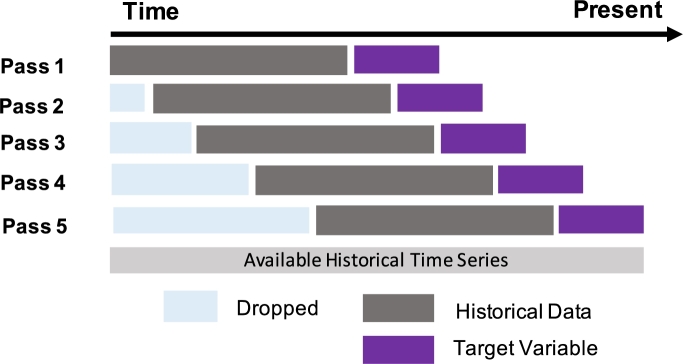
The sliding window backtesting method example used in this study for data pre-processing.

Firstly, we crawl data from ThaiDepression^2^ and transform them into proper CSV files for processing. Then, Fig. 3 illustrates how we create each sample input (*X*) and output (*Y*) using the sliding window back-testing approach. *X* represents the historical data with *n*-lag months, and *Y* represents the target variable, which is the future depression instances. In this study, *n*-lag months can vary from {1,2,3,4,5} to investigate how much past information we should use to predict future depression numbers in each area. The decision to use up to five months of lag was based on rigorous testing and analysis of the predictive performance across different lag durations. Our findings indicated that extending the lag beyond five months did not yield any significant improvement in the model's predictive accuracy. We separate data samples into two datasets where the first 90% is used for the training dataset and the last 10% is used for testing dataset to avoid data leakage in time series. Note that, due to its efficacy and simplicity in maintaining the statistical properties of the dataset, the mean imputation is used to address missing data. This technique preserves the overall mean, thereby minimizing bias in crucial statistical estimations.

Let *X*={$x_{1}$,$x_{2}$,...,$x_{t}$} be a multivariate time series sequence, with $x_{t}\in \{R\}$ representing all features at time t and $x_{t}^{i}\in \{R\}$ representing the value of the i−th feature seen at time *t*.

Since we both examine the performance of univariate and multivariate time series as well as the efficacy of machine learning and deep learning models in this context, we either denote $x_{t}^{i}$ as input time series for cities to predict the numbers of depression in a particular AHD or $x_{t}^{i}$ as input time series for AHDs to predict the numbers of depression in a country-level for multivariate time series (MTS) experiments. For the univariate time series (UTS) experiment, i is equal to 1 for $x_{t}^{i}$. We either denote $x_{t}^{i}$ as historical AHD time series to predict the future depression numbers in a particular AHD or $x_{t}^{i}$ can be framed as historical country-level depression profiles to forecast the future country-level depression profile. Then, *X* is normalized using the following Eq (1). As a consequence, all features are now ready to be used as input for further analysis.

(1) $$X=((X-X_{min}))/((X_{max}-X_{min}))$$

**Problem Definition:** We intend to examine models that can correctly anticipate future depression numbers at various administrative levels, including AHD-level and country-level, given a UTS and MTS training dataset *X*={$x_{1},x_{2},...,x_{t}$}.

### 3.2 Exploratory Data Analysis (EDA)

In this part, we present descriptive and statistical factor correlations pertaining to the geographical and temporal dynamics of the depression profile. We analyze the impact of historical *n*−lag univariate (UTS) and multivariate time series (MTS) data on monthly new depression numbers. We carry out the cross-correlation analysis with n-lag by selecting *n* between 1 and 5. The Pearson correlation coefficient [46] as shown in Eq (2) is used to calculate the correlation as follows.

(2) $$pearsonr=\frac{E[(A-\mu_{A})(B-\mu_{B})]}{\sqrt{\left(\Sigma_{i=1}^{N}{\left(A_{i}-\mu_{A}\right)}^{2}\right)}\sqrt{\left(\Sigma_{i=1}^{N}{\left(B_{i}-\mu_{B}\right)}^{2}\right)}}$$

where A = $\left[A_{1},...,A_{T}\right]$ and B = $\left[B_{1},...,B_{T}\right]$ are the two vectors that will be measured. We denote *N* as the time series length, and $\mu_{A}$ and $\mu_{B}$ denote the mean of A and B, respectively. The p-value and Pearson correlation coefficient are employed in the correlation analysis task. Only variables with a high correlation value and a p-value below 0.05 are considered statistically significant for further investigation. High correlations imply that historical administrative data may accurately reflect the national depression profile. As the cross-correlations on the subject of predicting national depressions have not yet been completely researched, the correlation with adjusting the *n*-lag is calculated.

### 3.3 Model training and testing

The objective function as shown in Eq (3) which is Mean Absolute Error (MAE) used for model optimization is as follows:

(3) $$arcmin(\Theta )=\Sigma_{T}|X_{i}-Y_{i}|$$

where Θ represents all trainable parameters in each model in the MONDEP framework, $X_{i}$ and $Y_{i}$ are actual and predicted values at sample *i*, and *T* is the number of the training samples in the dataset. The use of Mean Absolute Error (MAE) as our objective function ensures a reliable, straightforward measure of prediction accuracy in our depression forecasting framework. MAE effectively quantifies the average prediction errors, providing clear insights without being skewed by outliers. This makes it ideal for consistently evaluating model performance across diverse data sets and administrative regions, critical for effective public health strategies. Algorithm 1 displays the comprehensive learning algorithm.

**Algorithm 1:**
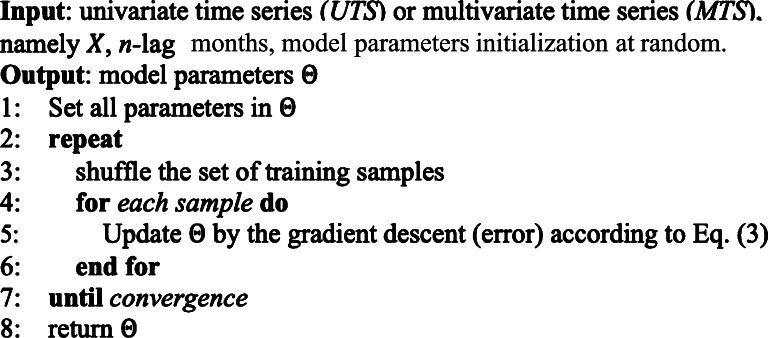
Learning algorithm for ST-MON framework.

## 4 Experimental setting

### 4.1 Dataset

**Figure 4:**
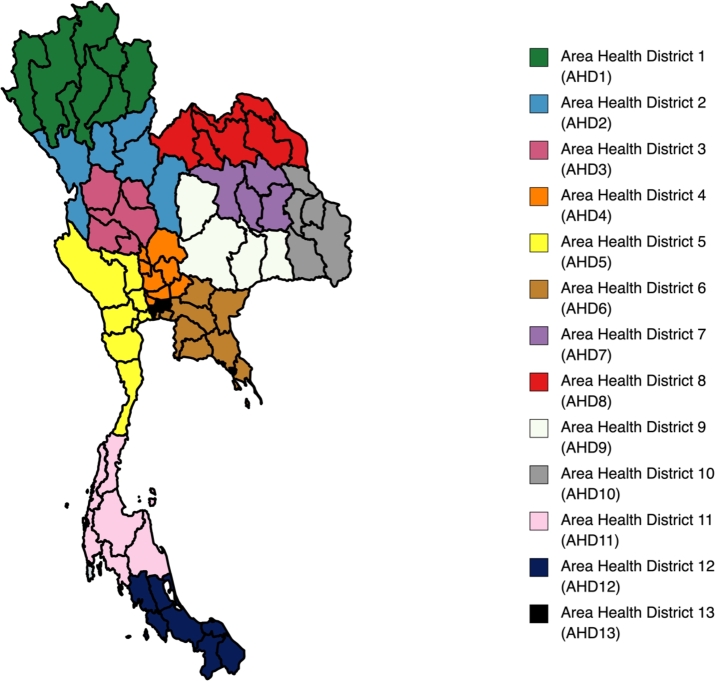
The visualization of Area Health District (AHD) from 1 to 13 in Thailand.

In order to examine the effects of various spatial and temporal factors on national depression forecasting in Thailand, we collect public statistical data on depression from https://thaidepression.com/www/report/main report/, which offers a report on the prevalence of depression in Thailand. The report provides a wide range of information regarding depression in Thailand, including its frequency, the demographics of those who suffer from it, and the most common types as shown in Fig. 4.

In this manuscript, the depression data utilized for analysis were collected over a period spanning from October 2016 to July 2022, with monthly sampling, resulting in a total of 70 data points. For each experiment conducted, with varying n-months lag as the time series input, the dataset was partitioned in a 90:10 ratio for training and testing purposes, respectively. For instance, in the 1-month lag experiment, statistical data from the previous month was employed to predict the current depression profile. In our experimental design, we have additionally implemented a *K*-fold cross-validation method where *K*=5 to improve the validity of our model's validation. By employing this methodology, we can conduct a more thorough assessment of the model's efficacy on various segments of the data, thereby guaranteeing that our conclusions are dependable on the larger dataset. A detailed explanation of the data sampling procedure can be found in Fig. 3. The raw datasets can be downloaded from.^3^

As shown in Fig. 4 and Table 2, Thailand has 13 Area Health Districts (AHDs), each with its own set of mental health services. These districts are located throughout the country and are responsible for providing mental health services to the local populations within their respective regions. Each AHD has its own set of mental health services, including hospitals, clinics, and community-based services. The mental health services offered by each AHD may vary depending on the needs of the local population, the resources available, and the priorities of the district. The descriptive statistics used in this study are shown in Table 3. We can see that there is a fluctuation in the number of depression cases across different AHDs. Overall, the mental health services offered by the 13 AHDs play a critical role in addressing the prevalence of depression in Thailand and improving the mental health of the local populations.

**Table 2:** Notation of Area Health District (AHD) and its cities.

Target Area	Sub-Area (Column)
Area Health District 1 (AHD1)	Chiang Rai, Nan, Phayao, Phrae, Chiang Mai, Mae Hong Son, Lampang, Lamphun
Area Health District 2 (AHD2)	Tak, Phitsanulok, Phetchabun, Sukhothai, Uttaradit
Area Health District 3 (AHD3)	Chai Nat, Kamphaeng Phet, Phichit, Nakhon Sawan, Uthai Thani
Area Health District 4 (AHD4)	Nonthaburi, Pathum Thani, Phra Nakhon Si Ayutthaya, Saraburi, Lopburi, Sing Buri, Ang Thong, Nakhon Nayok
Area Health District 5 (AHD5)	Kanchanaburi, Nakhon Pathom, Ratchaburi, Suphan Buri, Prachuap Khiri Khan, Phetchaburi, Samut Songkhram, Samut Sakhon
Area Health District 6 (AHD6)	Chachoengsao, Prachinburi, Sa Kaeo, Samut Prakan, Chanthaburi, Chonburi, Trat, Rayong
Area Health District 7 (AHD7)	Kalasin, Khon Kaen, Maha Sarakham, Roi Et
Area Health District 8 (AHD8)	Bueng Kan, Loei, Nong Khai, Nong Bua Lamphu, Udon Thani, Nakhon Phanom, Sakon Nakhon
Area Health District 9 (AHD9)	Chaiyaphum, Nakhon Ratchasima, Buriram, Surin
Area Health District 10 (AHD10)	Mukdahan, Yasothon, Si Sa Ket, Ubon Ratchathani, Amnat Charoen
Area Health District 11 (AHD11)	Chumphon, Nakhon Si Thammarat, Surat Thani, Krabi, Phang Nga, Phuket, Ranong
Area Health District 12 (AHD12)	Phatthalung, Trang, Narathiwat, Pattani, Yala, Songkhla, Satun
Area Health District 13 (AHD13)	Bangkok
Country Level	Area Health District 1 to 13

**Table 3:** Descriptive depression statistics of datasets used in experiments.

Area — Stat	mean	std	min	25%	50%	75%	max
AHD1	97221.53	29097.64	53650.00	64494.75	90658.50	124100.00	142732.00
AHD2	49751.53	11395.67	32594.00	43525.25	48046.00	59535.50	68142.00
AHD3	51697.74	12323.57	29595.00	41400.75	52569.50	62569.00	69140.00
AHD4	86375.90	21106.86	55602.00	67872.25	76781.00	106787.00	120106.00
AHD5	65859.97	12796.36	43044.00	54883.25	69784.50	76336.50	86057.00
AHD6	62619.67	15569.94	38746.00	47584.50	58178.00	77259.75	87629.00
AHD7	80294.00	14652.41	54437.00	68585.50	84586.50	92514.25	102001.00
AHD8	84006.41	12584.53	66020.00	73366.75	78281.50	96474.00	104396.00
AHD9	109713.79	23272.03	71525.00	88822.50	105637.50	130993.00	149595.00
AHD10	78230.60	18615.29	46691.00	58469.50	83622.50	94419.25	103588.00
AHD11	59776.76	13121.35	39516.00	49055.50	56686.00	72359.00	80413.00
AHD12	72503.36	13657.82	52629.00	60583.00	68965.00	85596.50	95622.00
AHD13	30983.30	5587.37	23228.00	26439.75	29669.50	35523.50	41324.00
Country_Level	929036.39	201451.54	607299.00	745528.75	903897.00	1114066.50	1250291.00

### 4.2 Baselines

We compare MONDEP with the following 12 baselines for evaluation:

1. SARIMAX [47]: It is also known as Seasonal Auto-Regressive Integrated Moving Average with eXogenous Factors, and is a model subclass of the ARIMA family. The moving-average term and the autoregressive term (AR) are intuitively the two components of ARIMA models (MA). The former sees the value at a given point in time as just the weighted sum of earlier values. The latter model represents the same value as a weighted sum of previous residuals instead.
2. Convolutional Neural Network (CNN) [48]: It is a type of artificial neural network or deep learning algorithm that can learn the appropriate weights and biases to distinguish the input characteristics from the others after receiving input. In order to learn specific filters or properties of specific inputs, it uses a mathematical technique known as convolution rather than universal matrix multiplication in at least one of its layers.
3. Recurrent Neural Network (RNN) [49]: It has a cycle formed by connections between nodes in this kind of artificial neural networks, allowing some nodes' output to influence other nodes' input in turn. It can display temporal dynamic behavior as a result of this.
4. Long Short Term Memory (LSTM) [50]: It is a kind of recurrent neural network that can collect order dependency in situations involving sequence prediction.
5. Gated Recurrent Units (GRU) [51]: It is a gating technique in recurrent neural networks that resembles a long short-term memory (LSTM) with fewer parameters.
6. Bidirectional LSTM (BiLSTM) [52]: It is a type of recurrent neural network architecture that processes input sequences in both forward and backward directions. It is especially helpful for applications like natural language processing, speech recognition, and handwriting recognition because it can collect context from past and future inputs. A Bidirectional LSTM divides the hidden layer into two distinct layers, one for processing data going forward and another for processing input going backward. The final result is created by concatenating the outputs of the two levels.
7. CNN with Attention [53]: It is a CNN with an attention network to learn the important features extracted by an attention network.
8. RNN with Attention [53]: It is an RNN with an attention network to learn the important features extracted by an attention network.
9. GRU with Attention [53]: It is a GRU with an attention network to learn the important features extracted by an attention network.
10. LSTM with Attention [53]: It is an LSTM with an attention network to learn the important features extracted by an attention network.
11. BiLSTM with Attention [53]: It is a Bi-directional LSTM with an attention network to learn the important features extracted by an attention network.
12. Time Series Transformer (TST) [54]: It is a Transformer architecture model to suit time series analysis. TST is customized for the sequential patterns inherent in time series data, including modifications in handling time-stamped data and a tailored attention mechanism.

### 4.3 Evaluation

We evaluate the performance of all models using the Mean Absolute Error (MAE), Mean Squared Error (MSE), Root Mean Squared Error (RMSE), and Mean Absolute Percentage Error (MAPE) on the testing dataset. Each metric measures the accuracy of the predictions, as shown in Eqs. (4)–(7).

**Mean Absolute Error (MAE):** It measures the discrepancies between observed and predicted values in samples. Normally, it is calculated by dividing the sample size by the sum of the absolute errors.

(4) $$MAE=\frac{1}{s}\Sigma_{i=1}^{s}|X_{i}-Y_{i}|$$

where $X_{i}$ and $Y_{i}$ are the actual and predicted values. *s* is total samples.

**Mean Square Error (MSE):** It is a calculation of the square root of the average error between the observed and predicted values in samples. Often, it is calculated by dividing the sample size by the sum of the absolute errors.

(5) $$MSE=\frac{1}{s}\Sigma_{i=1}^{s}{\left(X_{i}-Y_{i}\right)}^{2}$$

where $X_{i}$ and $Y_{i}$ are the actual and predicted values. *s* is total samples.

**Root Mean Square Error (RMSE):** The residualsśtandard deviation serves as a measure of how tightly the data are clustered around the model that fits the data the best. It is frequently used in regression analysis and forecasting to confirm the outcomes of experiments.

(6) $$RMSE=\sqrt{\frac{1}{s}\Sigma_{i=1}^{s}{\left(X_{i}-Y_{i}\right)}^{2})}$$

where $X_{i}$ and $Y_{i}$ are the actual and predicted values. *s* is total samples.

**Mean Absolute Percentage error (MAPE):** It is an accuracy measure that the formula expresses as a ratio.

(7) $$MAPE=\frac{1}{s}\Sigma_{i=1}^{s}\frac{\left|X_{i}-Y_{i}\right|}{\left|Y_{i}\right|}$$

where $X_{i}$ and $Y_{i}$ are the actual and predicted values. *s* is total samples.

### 4.4 Implementation detail

Mean Absolute Error (MAE) is used as the goal function in grid search to optimize the hyperparameters in the training process. We use the hyperparameter shown in Table 4, Table 5, Table 6 to train all models. The hyperparameter set we employ is the most effective mixture of several parameters, resulting in the lowest MAE. The optimal parameters of RNN, GRU, LSTM, BiLSTM in Table 4 are 16, 1, 16, 2, 5, Multi, 0.2 for number of hidden unit, number of layer, batch size, number of time step, patience, multivariate, and dropout, respectively. Also, the optimal parameters of RNN with Attention, GRU with Attention, LSTM with Attention, BiLSTM with Attention in Table 4 are 8, 1, 8, 2, 3, Multi, 0.2 for number of hidden unit, number of layer, batch size, number of time step, patience, multivariate, and dropout, respectively. Each experiment lasted 12 to 28 hours and used an Intel Xeon Platinum 8000 series (Skylake-SP) processor from the first generation. Python is used to implement our primary software. Also, you can see all of the details regarding our implementation at.^4^

**Table 4:** Hyperparameter for RNN, GRU, LSTM, BiLSTM, RNN with Attention, GRU with Attention, LSTM with Attention, BiLSTM with Attention.

Name	Hyperparameter
Number of Hidden Unit	8, 16, 32, 64, 128
Number of Layer	1, 2, 3
Batch Size	2, 4, 8, 16, 32, 64, 128
Number of Time Step	1, 2, 3, 4, 5
Patience	3, 5, 7, 10
Multivariate or Univariate Data	Multi, Uni
Dropout	0.2, 0.3, 0.5

**Table 5:** Hyperparameter grid-serach for CNN and CNN with Attention. The optimal parameters are bold.

Name	Hyperparameter
Filter Size	**16**, 32, 64, 128
Kernel Size	1, **2**, 3
Number of Layer	**1**, 2
Batch Size	2, 4, 8, 16, **32**, 64, 128
Number of Time Step	2, **3**, 4, 5
Patience	**3**, 5, 7, 10
Multivariate or Univariate Data	**Multi**, Uni

**Table 6:** Hyperparameter grid-serach for Time Series Transformer. The optimal parameters are bold.

Name	Hyperparameter
Total Dimension of the Model	128, **256**, 512
The Dimension of the	
Feedforward Network Model	**256**, 512, 1024
Number of Layer	**2**, 3, 4
Number of Epoch	10, **25**, 50, 100
Number of Time Step	1, **2**, 3, 4, 5
Multivariate or Univariate Data	**Multi**, Uni
Dropout	0.1, **0.2**, 0.4
Final Fully Connected Layer Dropout	0.0, **0.2**, 0.4

## 5 Experimental results

We outline the research questions in this part that we want to address through experimentation.

1. **RQ1.** How is the analysis and trend of national depression in Thailand?
2. **RQ2.** How is the relationship in terms of the number of depressions at the lower units to the higher units in a hierarchical structure?
3. **RQ3.** How do state-of-the-art models perform in national depression forecasting in Thailand, specifically, from the city to the district and from the district to the country?

### 5.1 Thai national depression analysis and trend (RQ1)

**Figure 5:**
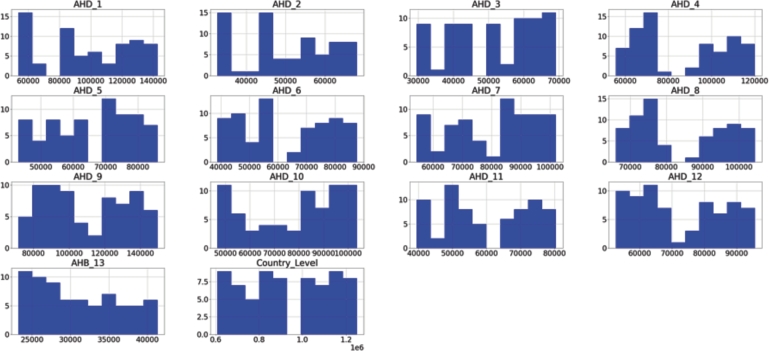
The distribution of the depression number throughout Thailand's Area Health Districts (AHDs) 1 through 13 and at the national level from October 2016 to July 2022.

In this part, we have looked into the characteristics of the various administrative levels of depression in Thailand. As illustrated in Fig. 5, we first look at the distribution of depression numbers across all levels. In Fig. 5, the frequency and number of depressions are shown by the *y* and *x*-axes, where the *x*-axis represents the frequency and the *y*-axis represents the bins of depression cases. The subsequent inferences can be drawn: 1) There is a binomial distribution (two modes) in the aggregate number of depressions from 2016 to 2022, particularly in AHD1, AHD3, AHD4, AHD5, AHD6, AHD8, AHD11, and country-level; 2) AHD4, AHD8, AHD9, and AHD10 have the highest number of depressions, with between 100,000 and 140,000 cases. This suggests that there is a peak period for people with mental health issues in Thailand's central and eastern regions at some point in time. Therefore, it motivates us to look into the cities in each AHD in this view.

**Figure 6:**
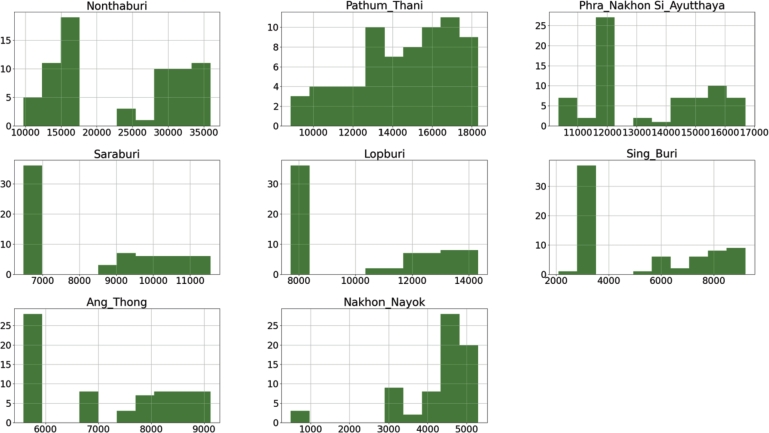
An illustration of the number of depressions distributed throughout all cities in AHD 4 from October 2016 to July 2022.

**Figure 7:**
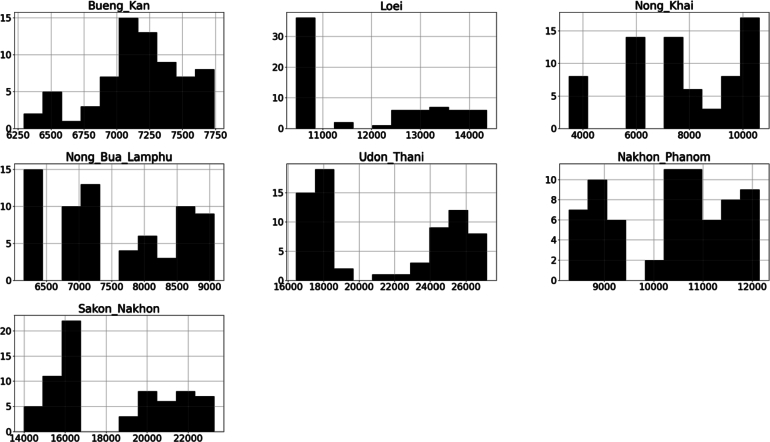
An illustration of the number of depressions distributed throughout all cities in AHD 8 from October 2016 to July 2022.

Figure 6, Figure 7 depict the distribution of depressions across all cities in AHD4 and AHD8, respectively. Nonthaburi, Pathum Thani, Phra Nakhon Si Ayutthaya, Saraburi, Lopburi, Sing Buri, Ang Thong, and Nakhon Nayok are the eight cities that belong to AHD4. Bueng Kan, Loei, Nong Khai, Nong Bua Lamphu, Udon Thani, Nakhon Phanom, and Sakon Nakhon are the eight cities that constitute AHD8. In the same AHD, we can see that the distribution of depression profiles varies among cities, but most follow the same trend and pattern. This suggests that certain AHDs may be represented by specific cities, and further investigation is required in the next section.

**Figure 8:**
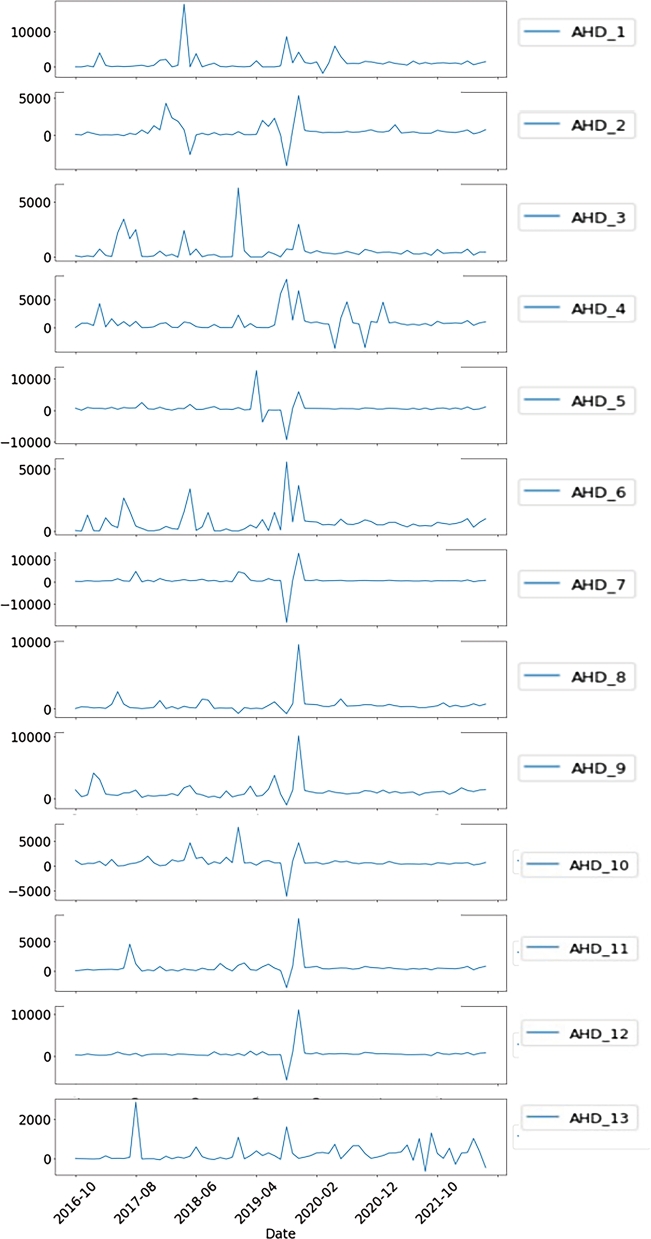
The trend of depression advancement at each location by computing the difference in the number of depression cases between the current month and the preceding month.

In order to look into the trend of depression advancement at each location, we compute the difference in the number of depression cases between periods $x_{t}$ (the current month) and $x_{t-1}$ (the preceding month), which is known as the $AHD_{delta}$. This enables us to examine the escalating and decreasing melancholy situations in the following months. The information in Fig. 8 allows us to conclude the following: 1) Between 2019 and 2020, while the pandemic is spreading globally, there is a sharp rise in the population of AHD6, AHD8, and AHD9 who have mental health issues. This suggests that these places are vulnerable and could require greater public mental health resources at a time when they're most needed; 2) Prior to the pandemic, there was a noticeable decrease in the number of people with mental illness in AHD2, AHD5, AHD7, AHD10, AHD11, and AHD12. Nevertheless, there has been a discernible rise in the prevalence of depression since the pandemic. This shows that there may be a higher risk of mental health issues throughout the post-pandemic eras. This is probably because stringent rules to stop the spread of the pandemic, such as work-from-home and isolation measures, have been put in place; 3) In AHD1, there was a noticeable rise in the number of patients with mental health conditions at the end of 2017. This was at the same time as Thailand's first significant issue with haze, smog, and PM2.5 pollution, notably in the country's north; 4) There are swings in the number of patients for AHD 3, AHD 4, and AHD 13, as well as a general upward and downward tendency. This suggests that there are unstable local events within these areas.

Based on the aforementioned findings, policymakers may coordinate the use of public mental health resources in reaction to specific events by keeping a close watch on the aforementioned trends.

### 5.2 Spatiotemporal depression analysis in a hierarchical structure (RQ2)

The Spatiotemporal Depression Analysis according to Eq. (2) is examined in this section. The Pearson correlation coefficients between monthly new depression cases and depression cases at various locations with *n*-month delays are shown in Table 7, where *n* is a number between 0 and 5. Table 7 demonstrates a substantial positive correlation (pearsonr > 0.9) between the population-level depression profile and its administrative depression profiles.

**Table 7:** Correlation Analysis between the depression numbers in country-level and its AHDs. The best performance on the representative n-day lag (Temporal Aspect) is in boldface, and The best performance on the representative location (Spatial aspect) is in parenthesis. All values have p-value <.00001.

	Pearson's correlation coefficient on n-days lag (Temporal Aspect)					
Location (Spatial Aspect)	0	1	2	3	4	5
AHD1	**0.9922**	0.9917	0.9910	0.9888	0.9866	0.9847
AHD2	**0.9899**	0.9880	0.9858	0.9864	0.9866	0.9861
AHD3	**0.9875**	0.9866	0.9860	0.9855	0.9854	0.9860
AHD4	0.9796	0.9800	**0.9809**	0.9794	0.9761	0.9727
AHD5	**0.9774**	0.9729	0.9692	0.9710	0.9730	0.9743
AHD6	0.9953	(0.9956)	**(0.9956)**	(0.9937)	(0.9920)	(0.9902)
AHD7	**0.9689**	0.9616	0.9550	0.9574	0.9600	0.9630
AHD8	**0.9880**	0.9850	0.9817	0.9790	0.9765	0.9737
AHD9	**0.9952**	0.9936	0.9919	0.9912	0.9908	0.9896
AHD10	0.9767	0.9759	0.9753	0.9771	0.9788	**0.9810**
AHD11	**(0.9954)**	0.9927	0.9897	0.9886	0.9878	0.9874
AHD12	**0.9939**	0.9896	0.9849	0.9834	0.9818	0.9805
AHD13	0.9864	0.9864	**0.9868**	0.9857	0.9844	0.9832

The following observations can be made based on Table 7. Firstly, AHD6 and AHD1 are the top two AHDs that can represent the national depression status as they have the highest pearsonr value. This confirms the notion that some AHDs can better represent the country's mental health status than others. Secondly, for each AHD, the current status of national mental health problems can be better captured with fewer *n*-month delays. On one hand, we observe that AHD1, AHD2, AHD3, AHD5, AHD7, AHD8, AHD9, AHD11, and AHD12 perform best when n=0, as indicated by their boldface values in Table 7. On the other hand, AHD4, AHD6, and AHD10 perform best at lag periods of 2 and 5, respectively. Lastly, the highest correlation value of 0.9956 belongs to AHD6, which represents the location around the country's eastern Gulf of Thailand coast and south of the provincial capital, at an n-lag period of 3. This suggests that in the past five years, the countermeasures taken in the AHD6 location have delayed impacting the national mental health status.

**Figure 9:**
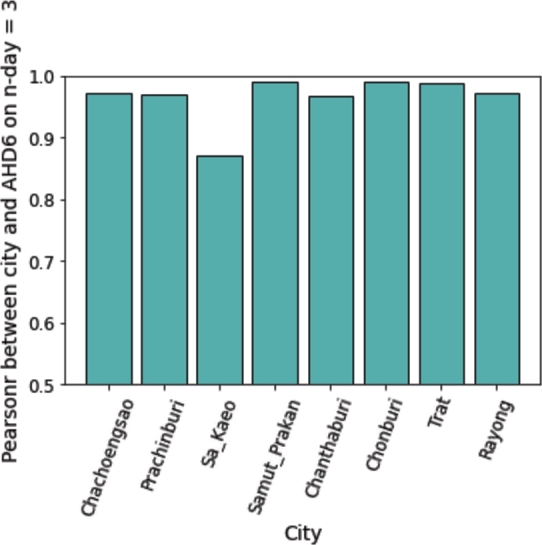
Correlation Analysis between the depression numbers in AHD6 and its cities. All values have p-value ≤ .00001.

The details of the correlation coefficient between AHD6 and its lower administrative cities with lag = 3 are illustrated in Fig. 9. Based on the statistical analysis, it can be inferred that Samut Prokan, Chonburi, and Trat are the cities that distinctly represent AHD6. At the same time, Sa Kaeo has a relatively weaker signal of the mental health problem at the upper administrative level. Based on the analysis, it can be concluded that there is a strong correlation between depression numbers in a hierarchical structure of administrative levels with respect to space and time.

Additionally, we have conducted Spearman's correlation analysis and applied the Bonferroni correction [55] between the two variables listed in Table 7 to address the risk of Type I errors. Our analysis shows a strong positive correlation with a Spearman's coefficient of 0.9 and a p-value near zero, leading us to reject the null hypothesis of no correlation. These methods ensure a more robust and accurate interpretation of the relationships in our complex health data.

### 5.3 Comparison of ML and DL models on depression forecasting (RQ3)

This section presents the results of the models in predicting depression status at both country-level and AHD-level. We use solely historical data for this work (univariate time series problem) or lower administrative-level data for the model's inputs (multivariate time series problem). For example, we may forecast future depression instances at AHD1 using historical data from AHD1 or historical AHD1's cities data. To assess the models' performance in different settings, experiments with over 100 model configurations were carried out, as shown in Table 8.

**Table 8:** Model settings.

Model	Algorithm	Uni/Multi-variate Time series	Temporal Lags
M_{1,2,3,4,5}	SARIMAX	Univariate	{1,2,3,4,5}
M_{6,7,8,9,10}	BiLSTM	Univariate	{1,2,3,4,5}
M_{11,12,13,14,15}	GRU	Univariate	{1,2,3,4,5}
M_{16,17,18,19,20}	LSTM	Univariate	{1,2,3,4,5}
M_{21,22,23,24,25}	RNN	Univariate	{1,2,3,4,5}
M_{26,27,28,29,30}	CNN	Univariate	{1,2,3,4,5}
M_{31,32,33,34,35}	BiLSTM with Attention	Univariate	{1,2,3,4,5}
M_{36,37,38,39,40}	GRU with Attention	Univariate	{1,2,3,4,5}
M_{41,42,43,44,45}	LSTM with Attention	Univariate	{1,2,3,4,5}
M_{46,47,48,49,50}	RNN with Attention	Univariate	{1,2,3,4,5}
M_{51,52,53,54,55}	CNN with Attention	Univariate	{1,2,3,4,5}
M_{56,57,58,59,60}	TST	Univariate	{1,2,3,4,5}
M_{61,62,63,64,65}	SARIMAX	Multivariate	{1,2,3,4,5}
M_{66,67,68,69,70}	BiLSTM	Multivariate	{1,2,3,4,5}
M_{71,72,73,74,75}	GRU	Multivariate	{1,2,3,4,5}
M_{76,77,78,79,80}	LSTM	Multivariate	{1,2,3,4,5}
M_{81,82,83,84,85}	RNN	Multivariate	{1,2,3,4,5}
M_{86,87,88,89,90}	CNN	Multivariate	{1,2,3,4,5}
M_{91,92,93,94,95}	BiLSTM with Attention	Multivariate	{1,2,3,4,5}
M_{96,97,98,99,100}	GRU with Attention	Multivariate	{1,2,3,4,5}
M_{101,102,103,104,105}	LSTM with Attention	Multivariate	{1,2,3,4,5}
M_{106,107,108,109,110}	RNN with Attention	Multivariate	{1,2,3,4,5}
M_{111,112,113,114,115}	CNN with Attention	Multivariate	{1,2,3,4,5}
M_{116,117,118,119,120}	TST	Multivariate	{1,2,3,4,5}

In Table 8, each model name is abbreviated as $M_{x}$, where *x* represents the number of temporal lags, and the model types, i.e., SARIMAX, BiLSTM, and time series types, i.e., univariate or multivariate, are denoted accordingly. Each row consists of five models, we select the best-performing model based on the lowest loss (MAE value) to represent specific experiments. These selected models are then compared in Table 9 to investigate the prediction of national depression, focusing on historical country data with a single variable and historical AHDs data with multiple variables. The objective is to discern the most effective model under different settings for predicting national depression, contributing to a better understanding of the predictive capabilities of the different model configurations.

**Table 9:** The model assessment for predicting national depressions is based either on historical country data with only one variable or on historical AHDs data with multiple variables. The boldface model is the finest. The model in brackets is the second-best one.

The Best Model	Algorithm	Time series	MAE	MSE	RMSE	MAPE
M_5	SARIMAX	Univariate	(518.3615)	(387,462.7211)	(622.4650)	(0.0037)
M_9	BiLSTM	Univariate	855.5000	1,650,234.5890	1,284.6146	0.0007
M_13	GRU	Univariate	1,027.4643	1,787,107.1380	1,336.8273	0.0008
M_19	LSTM	Univariate	797.9821	1,367,264.8500	1,169.3010	0.0006
M_21	RNN	Univariate	1,692.6250	4,786,319.2830	2,187.7658	0.0014
M_29	CNN	Univariate	1,264.8929	3,218,200.8130	1,793.9345	0.0010
M_35	BiLSTM with Attention	Univariate	13,457.839	238,082,193.00	15,429.9123	0.0110
M_40	GRU with Attention	Univariate	32,682.178	1,255,371,012.00	35,431.2152	0.0266
M_45	LSTM with Attention	Univariate	13,457.839	238,082,193.00	15,429.9123	0.0110
M_48	RNN with Attention	Univariate	13,110.696	239,453,352.60	15,474.2804	0.0107
M_53	CNN with Attention	Univariate	25,468.071	773,332,758.20	27,808.8612	0.0207
M_60	TST	Univariate	1,439.0579	3,459,360.2890	1,859.9356	0.0012
M_61	SARIMAX	Multivariate	750.7768	728,457.8801	853.4974	0.0054
M_66	BiLSTM	Multivariate	545.5714	514,087.3125	716.9988	0.0004
M_71	GRU	Multivariate	985.9464	1,669,362.6990	1,292.0382	0.0008
M_76	LSTM	Multivariate	545.5714	514,087.3125	716.9988	0.0004
M_82	RNN	Multivariate	593.8929	544,862.1696	738.1478	0.0005
M_87	CNN	Multivariate	**449.6786**	**278,893.6964**	**528.1039**	**0.0004**
M_91	BiLSTM with Attention	Multivariate	12,719.053	209,211,072.90	14,464.1306	0.0104
M_99	GRU with Attention	Multivariate	13,121.303	227,698,656.60	15,089.6871	0.0107
M_101	LSTM with Attention	Multivariate	12,719.053	209,211,072.90	14,464.1306	0.0104
M_106	RNN with Attention	Multivariate	12,806.678	229,564,882.60	15,151.3987	0.0105
M_115	CNN with Attention	Multivariate	13,269.928	239,166,174.40	15,464.9984	0.0108
M_118	TST	Multivariate	783.5162	1,048,961.2290	1,024.1881	0.0006

Table 9 shows the overall effectiveness of the four measurements as given in Eqs. (4)-(7). There are clear performance disparities across the models. Across all criteria for depression forecasting, the multivariate CNN model with time delays equal to 2 ($M_{87}$) performs better than all other models. The univariate SARIMAX model with time delays equal to 5 ($M_{5}$) and multivariate Bi-LSTM and LSTM models with time lags equal to 1 ($M_{66}$ and $M_{76}$), which have equivalent performance are the second- and third-best models, respectively. This supports our hypothesis that predicting the present state of the nation's mental-health problem using lower administrative-level historical data (AHDs) can better reflect the genuine situation from many different perspectives.

We can further make observations as follows. Firstly, we notice that a common model like SARIMAX performs best when applied to a univariate time series problem, indicating how simple the model is. In contrast, deep-learning models like LSTM, Bi-LSTM, TST, GRU, and CNN perform best when used to solve a multivariate time series problem, showcasing the model's ability to grasp the complex relationship between several signals. We also observe that univariate time-series models require longer temporal delays as model inputs than multivariate time-series models in order to achieve equivalent performance. Furthermore, we discovered that using deep learning models in conjunction with attention networks to solve the national depression forecasting problem in Thailand did not improve prediction accuracy. Generally, it is clear that utilizing multivariate time series models outperforms using univariate time series models in most cases.

The SARIMAX model, being a sophisticated extension of the classical ARIMA model, incorporates both seasonal and exogenous variables, which allows for a nuanced handling of univariate time series data. Its mathematical robustness lies in its ability to model and forecast time series data that exhibits non-stationary properties, a common characteristic in mental health data due to seasonal and circumstantial fluctuations. The SARIMAX model can be mathematically represented as follows in Eq (8):

(8) $$y_{t}=\mu +\phi_{1}y_{t-1}+\cdots +\phi_{p}y_{t-p}+\theta_{1}\epsilon_{t-1}+\cdots +\theta_{q}\epsilon_{t-q}+\beta X_{t}+\epsilon_{t}$$

where $y_{t}$ t is the variable being forecast, *μ* is the intercept, *ϕ* are the parameters for the autoregressive terms, *θ* are the parameters for the moving average terms, $\in_{t}$ are the error terms, *β* represents the coefficients of the exogenous inputs $X_{t}$, and *p*, *q* are the orders of the autoregressive and moving average parts of the model, respectively.

Conversely, the CNN model—a staple in deep learning applications—demonstrates its strength in handling multivariate time series data. This model leverages convolutional layers to process spatial hierarchies in data, allowing it to discern intricate patterns across multiple input variables simultaneously. The essential component of the CNN for time series data is the convolutional layer, which can be mathematically described by the convolution operation in Eq (9):

(9) $$\left(f⁎g)[n]=\sum_{m=-\infty }^{\infty }f[m]g[n-m\right]$$

where f(m) is the input signal (e.g., time series data), and g[n−m] is the kernel or filter applied to the input signal, capturing the temporal dependencies within the data at different scales. Both models, grounded in robust mathematical theories, contribute uniquely to our understanding and forecasting of depression. SARIMAX provides a solid foundation for traditional time series analysis, while CNN introduces advanced capabilities for dealing with multi-dimensional data structures. This dual approach allows us to address the complexities inherent in national mental health forecasting, resulting in more reliable and actionable insights.

In conclusion, it is evident that factors that are both geographical (data from different locations) and temporal (data from different time stamps) have a substantial influence in forecasting the country's mental health issue. We create a public-use visualization on Tableau Public to compare the mean absolute error (MAE) for all models based on different time steps and whether the data is univariate or multivariate for all experimental results in.^5^

### 5.4 The technical contributions

The multivariate CNN model with time delays of 2 ($M_{87}$) outperforms all others, followed by the univariate SARIMAX model with time delays of 5 ($M_{5}$), and the multivariate Bi-LSTM and LSTM models with time lags of 1 ($M_{66}$ and $M_{67}$), which demonstrate equivalent performance as the second and third-best models, respectively. This supports the hypothesis that utilizing lower administrative-level historical data (AHDs) for predicting the nation's mental health can better reflect the genuine situation from multiple perspectives. Notably, univariate time-series models like SARIMAX excel in univariate tasks, while deep-learning models such as LSTM, Bi-LSTM, TST, GRU, and CNN shine in multivariate scenarios, capturing complex signal relationships. Multivariate time-series models outperform univariate models in most cases, indicating their superiority for this forecasting problem in Thailand. Additionally, using attention networks alongside deep learning models did not improve prediction accuracy in this context.

## 6 Discussion on depression and suicidal tendency correlation analysis

Depression is a serious mental health condition that can significantly impact a person's mood, thoughts, and behavior. While not all individuals with depression will experience suicidal ideation or engage in suicidal behaviors, depression is a major risk factor for suicide. There are many pieces of evidence show how depression and suicide are related. For example, [56], [57], [58] Previous studies found patients with depression were 20 times more likely to attempt suicide compared to others without depression. Specifically, the authors discovered that persons with depression were 20 times more likely to attempt suicide than those without depression.

**Figure 10:**
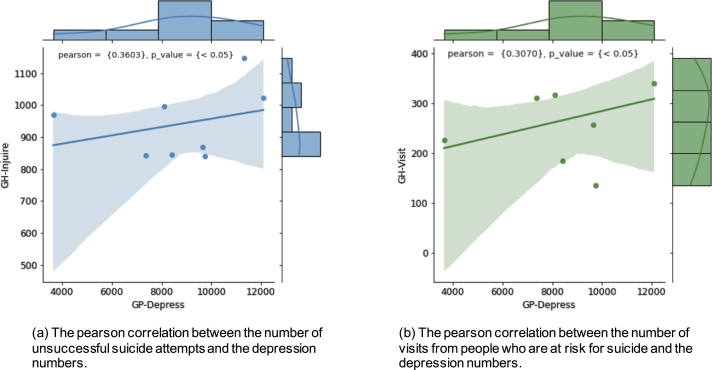
Correlation Analysis between the depression numbers and suicidal indicators in Thailand.

The indication of suicidal inclinations from depression trends in Thailand is thus studied in this section. We make use of the Thailand statistics datasets from [59] to ascertain the relationship between suicidal ideation and depression cases. As seen in Fig. 10 (a) and (b), the depressed signal and those who have attempted or are considering suicide are positively correlated with GH-Visit and GH-Injure with pearsonr values of around 0.3603 and 0.3070, respectively. GH-Visit denotes the number of visits from people who are at risk for suicide, whereas GH-Injure denotes the number of unsuccessful suicide attempts. Overall, the results show that depression is a significant risk factor for suicide and that receiving effective therapy may significantly reduce the chance of engaging in suicidal behavior. Given that depression is a medical condition that can be treated, it is critical for those experiencing depressed symptoms to seek assistance from a mental health professional.

## 7 Discussion on generalizability across different healthcare systems

The MONDEP framework, which was originally proven in Thailand, may be used to various locations with different healthcare systems and diverse data availability. In order to investigate this matter, we have included data from the United States,^6^ especially utilizing state depression profiles from Mental Health America to forecast the nation's comprehensive depression profile. This methodology enables us to evaluate the flexibility of our methodologies, originally designed for the healthcare system of Thailand, in a markedly distinct setting such as the United States.

For a real-world implementation of this idea, we employed our best model, a Convolutional Neural Network (CNN) with a lag of 2, which was first trained using data from Thailand. Subsequently, this model was employed to assess its efficacy within the context of the United States healthcare system. The outcomes of this interregional application, encompassing an intricate evaluation of performance between Thailand and the USA, are exhibited in Table 10. Remarkably, the outcomes from all indicators are similar, suggesting that our approach is resilient in forecasting the prevalence of depression cases at the population level. This discovery is noteworthy as it implies the capability of our model to be modified for utilization in various healthcare environments without sacrificing accuracy.

**Table 10:** The model assessment for predicting national depressions in Thailand and USA based on the best model, which is CNN with a lag of 2.

Algorithm	Time series	MAE	MSE	RMSE	MAPE
CNN with Thailand depression dataset	Multivariate	449.6786	278,893.6964	528.1039	0.0004
CNN with USA depression dataset	Multivariate	127.0313	16,136.9385	127.0312	0.0002

## 8 Discussion on practical implications and real-world applications

The results from our study highlight the significant real-world applications of the MONDEP framework, demonstrating its immense potential to serve as a crucial tool for public health policymakers and healthcare providers. By accurately forecasting national depression trends, the framework allows policymakers to strategically allocate resources and implement interventions in high-risk areas more effectively. Healthcare providers can utilize this tool to improve mental health services and offer specialized support to those most in need. Additionally, the capability of the framework to continuously track and analyze depression patterns equips professionals with the means to swiftly identify emerging trends and potential crises in mental health. Such proactive surveillance supports the early deployment of preventive strategies and interventions, significantly reducing the adverse effects of depression on individuals and broader communities. Ultimately, the findings from this study provide a robust foundation for enhancing population-wide mental health strategies, equipping stakeholders with a dynamic tool for informed decision-making and efficient management of mental health services.

## 9 Conclusion

In this study, we proposed the MONDEP hierarchical spatial-temporal framework to forecast future depressive patients in Thailand. Our unique perspective imitates the method of aggregating depression statistics from different administrative levels. We conducted several experiments to determine the optimal geographic and temporal parameters for depression forecasting. We found that the most accurate forecasts were achieved using multivariate time series data and a deep-learning model like a convolution neural network. To support upcoming research on national depression modeling in other nations, we have released our open-source source code. We believe that our technique might assist policymakers in managing mental health concerns by providing a quantitative and in-depth investigation of the impact of multivariate time-series variables on depression transmission in almost real-time. Looking ahead, we plan to enhance the MONDEP framework by incorporating additional contextual factors such as news trends, stock market fluctuations, employment rates, and other relevant socio-economic indicators. This expansion aims to provide a more holistic understanding of the factors influencing national mental health. Additionally, we plan to include more social media and other socioeconomic data in our system to increase our ability to anticipate the state of the nation's mental health in the future. By broadening the scope of our data inputs, we aim to refine our forecasting capabilities and offer more comprehensive tools for policymakers and researchers in the field of mental health.

## Ethics declarations

These statements affirm that our research has been conducted ethically, with careful attention to privacy and consent. The dataset used in this study is publicly available, ensuring transparency and accessibility in our research practices.

## CRediT authorship contribution statement

**Tipajin Thaipisutikul:** Writing – review & editing, Writing – original draft, Visualization, Validation, Supervision, Project administration, Methodology, Formal analysis, Data curation, Conceptualization. **Pasinpat Vitoochuleechoti:** Validation, Methodology, Data curation. **Papan Thaipisutikul:** Writing – original draft, Investigation. **Suppawong Tuarob:** Resources, Investigation, Formal analysis, Conceptualization.

## Declaration of Competing Interest

The authors declare that they have no known competing financial interests or personal relationships that could have appeared to influence the work reported in this paper.

## Data Availability

The data supporting the findings of this study are available at https://github.com/TipGreenTea/ThailandDepression and bit.ly/3Y9uH8N.

## Footnotes

1 https://github.com/TipGreenTea/ThailandDepression.

## Footnotes

2 https://thaidepression.com/www/report/main_report/.

3 bit.ly/3Y9uH8N.

4 https://github.com/TipGreenTea/ThailandDepression.

5 https://public.tableau.com/app/profile/pasinpat.vitoochuleechoti1646/viz/AllResult/Story1.

6 https://mhanational.org/mhamapping/mha-state-county-data.

## References

[1] Gbiri C.A., Akinpelu A.O., Odole A.C. (2010). Prevalence, pattern and impact of depression on quality of life of stroke survivors. Int. J. Psychiatry Clin. Pract..

[2] Meaklim H., Junge M.F., Varma P., Finck W.A., Jackson M.L. (2021). Pre-existing and post-pandemic insomnia symptoms are associated with high levels of stress, anxiety, and depression globally during the covid-19 pandemic. J. Clin. Sleep Med..

[3] Birtel M.D., Wood L., Kempa N.J. (2017). Stigma and social support in substance abuse: implications for mental health and well-being. Psychiatry Res..

[4] Chavous T.M., Molock S.D., Sanchez-Parkinson L. (2022). Introduction: mental health among marginalized communities. Curr., J. Divers. Scholarsh. Soc. Change.

[5] Bhagavathi P., Vitone T. (2022). Analysis of adolescent barriers in seeking help for mental health issues. J. Stud. Res..

[6] Knapp M. (2020). New Oxford Textbook of Psychiatry.

[7] Hong S., Walton B., Kim H.-W., Rhee T.G. (2023). Predicting the behavioral health needs of Asian Americans in public mental health treatment: a classification tree approach. Adm. Policy Ment. Health Mental Health Serv. Res..

[8] Gao S., Calhoun V.D., Sui J. (2018). Machine learning in major depression: from classification to treatment outcome prediction. CNS Neurosci. Ther..

[9] Baraka A.A., Ramadan F.H., Hassan E.A. (2021). Predictors of critical care nurses' stress, anxiety, and depression in response to covid-19 pandemic. Nurs. Crit. Care.

[10] Colledani D., Anselmi P., Robusto E. (2023). Development of a scale for capturing psychological aspects of physical–digital integration: relationships with psychosocial functioning and facial emotion recognition. AI Soc..

[11] Verma V., Saini P., Priyanshu, Nischal A. (2022). Session-based recommendation using recurrent neural networks: a comparative theoretical analysis. Authorea.

[12] Ying H., Zhuang F., Zhang F., Liu Y., Xu G., Xie X., Xiong H., Wu J. (2018). Proceedings of the Twenty-Seventh International Joint Conference on Artificial Intelligence.

[13] Zheng S., Gao Z., Cao W., Bian J., Liu T.-Y. (2021). Proceedings of the 30th ACM International Conference on Information & Knowledge Management.

[14] Pei S., Shaman J. (2020). Initial simulation of sars-cov2 spread and intervention effects in the continental us. 10.1101/2020.03.21.20040303.

[15] Saeed U. (2022). The economic repercussions of coronavirus disease 2019 (covid-19). Coronavirus Dis..

[16] Aral N., Bakir H. (2022). Spatiotemporal analysis of covid-19 in Turkey. Sustain. Cities Soc..

[17] Welegedara N.P., Agrawal S.K., Lotfi G. (2023). Exploring spatiotemporal changes of the urban heat island effect in high-latitude cities at a neighbourhood level: a case of Edmonton, Canada. Sustain. Cities Soc..

[18] Metzger M.-H., Tvardik N., Gicquel Q., Bouvry C., Poulet E., Potinet-Pagliaroli V. (2016). Use of emergency department electronic medical records for automated epidemiological surveillance of suicide attempts: a French pilot study. Int. J. Methods Psychiatr. Res..

[19] Leroy G., Gu Y., Pettygrove S., Galindo M.K., Arora A., Kurzius-Spencer M. (2018). Automated extraction of diagnostic criteria from electronic health records for autism spectrum disorders: development, evaluation, and application. J. Med. Internet Res..

[20] He Q., Veldkamp B.P., Glas C.A., de Vries T. (2016). Automated assessment of patients' self-narratives for posttraumatic stress disorder screening using natural language processing and text mining. Assessment.

[21] Goodwin T.R., Maldonado R., Harabagiu S.M. (2017). Automatic recognition of symptom severity from psychiatric evaluation records. J. Biomed. Inform..

[22] Cook B.L., Progovac A.M., Chen P., Mullin B., Hou S., Baca-Garcia E. (2016). Novel use of natural language processing (nlp) to predict suicidal ideation and psychiatric symptoms in a text-based mental health intervention in Madrid. Comput. Math. Methods Med..

[23] Pestian J.P., Grupp-Phelan J., Bretonnel Cohen K., Meyers G., Richey L.A., Matykiewicz P., Sorter M.T. (2015). A controlled trial using natural language processing to examine the language of suicidal adolescents in the emergency department. Suicide Life-Threat. Behav..

[24] Lin S., Wu Y., Fang Y. (Jan 2022). Comparison of regression and machine learning methods in depression forecasting among home-based elderly Chinese: a community based study. Front. Psychiatry.

[25] Zhou Z., Luo D., Yang B.X., Liu Z. (Jan 2022). Machine learning prediction models for depression symptoms among Chinese health care workers during the covid-19 outbreak. 10.2196/preprints.36814.

[26] Iyortsuun N.K., Kim S.-H., Jhon M., Yang H.-J., Pant S. (2023). A review of machine learning and deep learning approaches on mental health diagnosis. Healthcare.

[27] Aleem S., Huda N.u., Amin R., Khalid S., Alshamrani S.S., Alshehri A. (2022). Machine learning algorithms for depression: diagnosis, insights, and research directions. Electronics.

[28] Chung J., Teo J. (Jan 2023). Single classifier vs. ensemble machine learning approaches for mental health prediction. Brain Inform..

[29] Janacek G. (2010). Time series analysis forecasting and control. J. Time Ser. Anal..

[30] Shao Z. (2022). 2022 14th International Conference on Machine Learning and Computing (ICMLC).

[31] Montero-Manso P., Athanasopoulos G., Hyndman R.J., Talagala T.S. (2020). Fforma: feature-based forecast model averaging. Int. J. Forecast..

[32] Oukhouya H., Kadiri H., El Himdi K., Guerbaz R. (2023). Forecasting international stock market trends: xgboost, lstm, lstm-xgboost, and backtesting xgboost models. Stat. Optim. Inf. Comput..

[33] Hochreiter S., Schmidhuber J. (1997). Long short-term memory. Neural Comput..

[34] Puszkarski B., Hryniów K., Sarwas G. (2022). Comparison of neural basis expansion analysis for interpretable time series (n-beats) and recurrent neural networks for heart dysfunction classification. Physiol. Meas..

[35] Wu Z., Pan S., Long G., Jiang J., Chang X., Zhang C. (2020). Proceedings of the 26th ACM SIGKDD International Conference on Knowledge Discovery & Data Mining.

[36] Wan R., Mei S., Wang J., Liu M., Yang F. (2019). Multivariate temporal convolutional network: a deep neural networks approach for multivariate time series forecasting. Electronics.

[37] Wang J.-F., Christakos G., Han W.-G., Meng B. (2008). Data-driven exploration of ‘spatial pattern-time process-driving forces’ associations of sars epidemic in Beijing, China. J. Public Health.

[38] Bourdin S., Jeanne L., Nadou F., Noiret G. (2021). Does lockdown work? A spatial analysis of the spread and concentration of covid-19 in Italy. Reg. Stud..

[39] Wang P., Li Y., Pan Z. (2021). Proceedings of the 1st International Conference on Public Management and Big Data Analysis.

[40] Chen Y.-C., Thaipisutikul T., Shih T.K. (2022). A learning-based poi recommendation with spatiotemporal context awareness. IEEE Trans. Cybern..

[41] Boppuru P.R., Ramesha K. (2020). Spatio-temporal crime analysis using KDE and Arima models in the Indian context. Int. J. Digit. Crime Forensics.

[42] Prathap B.R., Ramesha K. (2020). Geospatial crime analysis to determine crime density using kernel density estimation for the Indian context. J. Comput. Theor. Nanosci..

[43] Jasny L., Long S., Minihane J., Wu J. (2022).

[44] Prathap B.R. (2023). Geo-spatial crime density attribution using optimized machine learning algorithms. Int. J. Inf. Technol..

[45] Prathap B.R., Krishna A.V., Balachandran K. (2021). Crime analysis and forecasting on spatio temporal news feed data—an Indian context. Stud. Big Data.

[46] Thaipisutikul T., Lin C.-Y., Chen S.-C. (2022). 2022 19th International Joint Conference on Computer Science and Software Engineering (JCSSE).

[47] Fiskin C.S., Guldem Cerit A. (2019). 2019 4th International Conference on Computer Science and Engineering (UBMK).

[48] Véstias M.P. (2022). Convolutional neural network. Res. Anthology Artif. Neural Netw. Appl..

[49] Ahsan N., Ayyad M., Hajj M., Akhtar I. (2023). AIAA SCITECH 2023 Forum.

[50] Osman O., Rakha H., Mittal A. (2021). Application of long short term memory networks for long- and short-term bus travel time prediction. 10.20944/preprints202104.0269.v1.

[51] Rehmer A., Kroll A. (2020). On the vanishing and exploding gradient problem in gated recurrent units. IFAC-PapersOnLine.

[52] Biswas S. (2022). 2022 1st International Conference on AI in Cybersecurity (ICAIC).

[53] Shao Z. (2022). 2022 14th International Conference on Machine Learning and Computing (ICMLC).

[54] Li Z., Zhang X., Dong Z. (2022). Tsf-transformer: a time series forecasting model for exhaust gas emission using transformer. Appl. Intell..

[55] McCloskey A. (2012). Bonferroni-based size-correction for nonstandard testing problems. SSRN Electron. J..

[56] Omary A. (2020). National prevalence rates of suicidal ideation and suicide attempts among adults with and without depression. J. Nerv. Ment. Dis..

[57] Quiñones V., Jurska J., Fener E., Miranda R. (2015). Active and passive problem solving: moderating role in the relation between depressive symptoms and future suicidal ideation varies by suicide attempt history. J. Clin. Psychol..

[58] AbdElmageed R.M., Mohammed Hussein S.M. (2022). Risk of depression and suicide in diabetic patients. Cureus.

[59] Noraset T., Chatrinan K., Tawichsri T., Thaipisutikul T., Tuarob S. (2022). Language-agnostic deep learning framework for automatic monitoring of population-level mental health from social networks. J. Biomed. Inform..

